# Impact of Fruit Piece Structure in Yogurts on the Dynamics of Aroma Release and Sensory Perception

**DOI:** 10.3390/molecules18056035

**Published:** 2013-05-21

**Authors:** Joshua Mesurolle, Anne Saint-Eve, Isabelle Déléris, Isabelle Souchon

**Affiliations:** 1 INRA, UMR 782 Génie et Microbiologie des Procédés Alimentaires, 1 avenue Lucien Brétigniéres, 78850 Thiverval-Grignon, France; E-Mails: joshua.mesurolle@grignon.inra.fr (J.M.); isabelle.deleris@grignon.inra.fr (I.D.); isabelle.souchon@grignon.inra.fr (I.S.); 2 AgroParisTech, UMR 782 Génie et Microbiologie des Procédés Alimentaires, 1 avenue Lucien Brétigniéres, 78850 Thiverval-Grignon, France

**Keywords:** dairy gels, fruit piece hardness, descriptive profile, temporal dominance of sensations, flavor release, proton transfer reaction-mass spectrometry

## Abstract

The aim of this work was to gain insight into the effect of food formulation on aroma release and perception, both of which playing an important role in food appreciation. The quality and quantity of retronasal aroma released during food consumption affect the exposure time of olfactory receptors to aroma stimuli, which can influence nutritional and hedonic characteristics, as well as consumption behaviors. In yogurts, fruit preparation formulation can be a key factor to modulate aroma stimulation. In this context, the impact of size and hardness of fruit pieces in fat-free pear yogurts was studied. Proton Transfer Reaction-Mass Spectrometry (PTR-MS) was used to allow sensitive and on-line monitoring of volatile odorous compound release in the breath during consumption. In parallel, a trained panel used sensory profile and Temporal Dominance of Sensations (TDS) methods to characterize yogurt sensory properties and their dynamic changes during consumption. Results showed that the size of pear pieces had few effects on aroma release and perception of yogurts, whereas fruit hardness significantly influenced them. Despite the fact that yogurts presented short and similar residence times in the mouth, this study showed that fruit preparation could be an interesting formulation factor to enhance exposure time to stimuli and thus modify food consumption behaviors. These results could be taken into account to formulate new products that integrate both nutritional and sensory criteria.

## 1. Introduction

Many types of stirred yogurts are present on the market, varying in fat, sugar, texture, flavor and types of fruit. Faced with this extensive range of products, sensory characteristics are crucial for product acceptance and consumer pleasure [1,2]. Sensory characteristics were notably identified as one of the main factors responsible for the purchase of low-calorie yogurts [3].

Due to the nutritional recommendations of public authorities and current health issues, particularly in terms of overweight and obesity, consumers are increasingly vigilant with regard to food product qualities. In this context, low-fat yogurts fulfill consumer expectations in terms of nutritional quality, but are often less appreciated than standard ones from a sensory point of view. Improving the sensory quality of this kind of food products is thus a real challenge for the food industry, but requires a better understanding of the impact of product composition and structure on aroma release at the origin of perception. Some studies have highlighted the fact that increasing aroma release and exposure time to stimuli could play a role on satiety and food intake [4]. It would be interesting to consider this result as a complementary solution to better formulate flavored food products in agreement with nutritional recommendations and consumer expectations.

Among all perceptions that occur during the dynamic process of food consumption, aroma perception plays a considerable role, particularly in the case of products with a short residence time in the mouth. In order to improve aroma perception, a better understanding of the factors that can influence in-mouth aroma release is of major importance [5,6]. In recent years, different instrumental and sensory techniques have been developed to address this issue. Regarding aroma release while eating, nose-space methods such as atmospheric pressure chemical ionization mass spectrometry (APCI-MS) and proton transfer reaction mass spectrometry (PTR-MS) are frequently used [7,8,9]. To study dynamic perceptions, techniques such as Time-Intensity or Temporal Dominance of Sensations are applied more and more often [10,11,12,13]. Finally, numerous studies have addressed the relationships between aroma release and aroma perception [14,15,16,17,18]. On the basis of these studies, it was shown that aroma perception was influenced not only by the amount of aroma compounds released, but also by the release rate and the shape of the release curve during consumption [19]. This kind of approach seems to be well adapted to investigate the exposure time to stimuli and to understand the role of some formulation factors on aroma release at the origin of perception.

Even if aroma perception results from the combination of complex physicochemical, biochemical, physiological, psychological and neurobiological phenomena [20,21], the main role of food product composition [22,23,24,25,26,27] and its structure [8,13,28,29,30], as well as cognitive phenomena [5,31], have been highlighted in the case of dairy products. The important role of intra-oral manipulations [5,32,33,34] has also been demonstrated in many studies.

Yogurt is a semi-solid dairy product that requires very little oral movement before swallowing: it is diluted in saliva and almost directly transported from the front of the mouth to the oropharynx, mainly by tongue movements against the palate [35]. Given the impact of residence time and intra-oral manipulations of a product in the mouth on aroma release, the addition of fruit preparations in yogurts could help to modify oral behavior. Changing the rheological parameters of fruit preparations in yogurts by adding thickeners seems to play a minor role on aroma release, compared to the composition of fruit preparations [25]. However, as far as we know, the impact of the structure of fruit pieces within fruit preparations has never been investigated. Concerning oral behavior adaptation when heterogeneous foods are eaten, it was shown that the size and concentration of nuts in yogurts influence the swallow threshold [36]. Even if fruit pieces are softer than nuts, their structural characteristics such as size and hardness could still have an impact on oral behavior and modify product residence time, for example. In this context, the present study proposes to study the impact of some characteristics of fruit pieces on aroma release and perception in the case of flavored yogurts. 

## 2. Results and Discussion

### 2.1. Physico-Chemical Characterization of Yogurts

Hardness of pear pieces in fruit preparations was measured by a puncture test performed at the GMPA laboratory with a TA-XT2 texture analyzer. Results are presented in Table 1 and show that hard pieces were two times harder than soft pieces. Hardness of pear pieces was not measured in the case of P_Size pear pieces.

**Table 1:** Characteristics of fruit pieces in fruit preparations used in the three different yogurts.

Yogurt name	Fruit size (mm)	Fruit hardness (N)	Pear variety
P_Standard	6	0.142 ± 0.117	Rocha
P_Size	10	Not measured	Rocha
P_Hardness	6	0.358 ± 0.215	Williams

The rheological properties and pH of yogurts were measured at each sensory session to monitor product quality and to verify their stability over time. The viscoelastic properties of yogurts, measured by an oscillatory test performed with a MCR301 rheometer at a frequency varying from 10 to 0.1 Hz and with a constant deformation of 1%, showed that the mean storage and loss moduli for the three yogurts were G' = 1.20 10^3^ ± 651 Pa and G'' = 3.13 10^2^ ± 151 Pa, respectively, and remained stable throughout the test period. The mean pH of products, measured at 4 °C with a pH-metric probe, was 4.4 ± 0.2 and also remained stable throughout the test period.

### 2.2. Selection of Ions to be Monitored and Contribution of Pear Preparations to the Total Aroma Release

An *in vitro* study was first performed to study the fragmentation pattern of yogurt main aroma compounds. Single molecules present in aroma formula used in fruit preparations were diluted in water and the headspace above the solution was analyzed using a High Sensitive Proton Transfer Reaction Mass Spectrometer (PTR-MS) in scan mode, as described in the Experimental Section. The fragmentation patterns are presented in Table 2.

**Table 2:** Fragmentation patterns of yogurt main aroma compounds, determined during *in vitro* measurements by PTR-MS.

Aroma compound	Main fragment (*m/z*)	Other fragments (% of main fragment intensity)							
Hexyl acetate	61	43(78)	41(27)	39(14)	57(5)	85(4)	62(3)	44(2)	145(1)
Isoamyl acetate	41	43(83)	39(53)	71(30)	61(6)	42(3)	44(3)	55(2)	27(1)
Octyl acetate	43	61(95)	41(88)	57(63)	39(42)	71(14)	42(6)	58(3)	
Ethyl acetate	61	43(82)	89(14)	44(2)					
γ-decalactone	47	61(99)	43(99)						
*cis*-3-Hexenyl acetate	55	83(46)	39(13)	43(7)	53(6)	61(5)	56(5)	84(3)	29(3)
Geranyl butyrate	55	41(99)	39(50)	83(50)					
Furaneol	61	92(87)	43(77)	45(28)	91(30)	72(8)			
Diacetyl	87								
Anisaldehyde	137	33(16)	109(16)						

The two fruit preparations with different pear varieties were also individually analyzed under *in vitro* conditions using PTR-MS instrument in scan mode, as described in the Experimental Section. An additional *in vitro* study was performed to study the contribution of pear preparations to the total aroma release and to select specific ions to be measured during *in vivo* measurements. The headspaces above yogurts with and without fruit preparation and aroma were analyzed with the same protocol. Molecule fragments that made it possible to distinguish products and that could be related to aroma molecules were preferentially chosen (Table 3). 

**Table 3:** Description of the fragments (*m/z*) monitored during *in vivo* measurements by PTR-MS, the main molecules from which they are derived and the main sensory notes associated with them.

Ion (*m/z*)	Main molecules related to fragments (determined from *in vitro* study data)	Sensory notes [37]
55	*cis*-3-Hexenyl acetate	Fruity, fresh green
61	Esters *, Furaneol	Fruity pear, caramel
71	Isoamyl acetate, octyl acetate	Banana, fruity
72	Furaneol	Caramel, cooked fruits
87	Diacetyl	Butter
137	Anisaldehyde	Anis

* Hexyl acetate, ethyl acetate, octyl acetate

**Figure 1:**
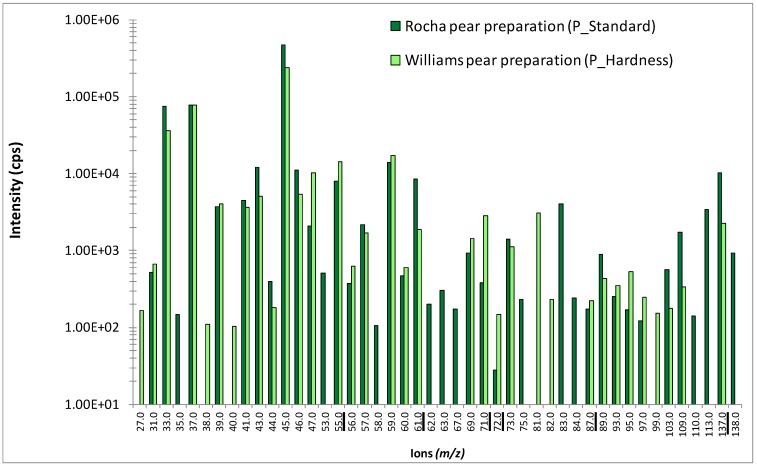
Comparison of PTR-MS spectra obtained from *in vitro* measurements performed in the headspace of the two pear preparations that varied in pear piece hardness (logarithmic scale). Peaks are labeled by their *m/z* ratios, and their intensity was determined by PTR-MS in scan mode. Underlined ions were selected for the *in vivo* study. The PTR-MS instrument drift tube was thermally controlled (60 °C) and operated with voltage and pressure set at 600.1 (± 0.4) V and 1.9 (± 0.06) mbar, respectively.

Figure 1 presents PTR-MS spectra obtained during *in vitro* measurements in the headspace above Rocha and Williams pear preparations. Many fragments were common to the two pear varieties (28 out of 46), and the highest peaks presented a similar intensity for both Rocha and Williams pear preparations (*m/z* 33, *m/z* 43, *m/z* 45, *m/z* 46, *m/z* 55 and *m/z* 87). However, some ions were specific to the Rocha pear preparation or released with the highest intensity from this preparation (for example, fragments *m/z* 53, *m/z* 61, *m/z* 83, *m/z* 113 and *m/z* 137), whereas others were specific to the Williams pear variety or released with the highest intensity from this last preparation (for example, fragments *m/z* 27, *m/z* 71, *m/z* 72 and *m/z* 81). 

Concerning *in vivo* measurements, panelists’ breath was analyzed during yogurt consumption with the Multiple Ion Detection mode of PTR-MS instrument. On the basis of previous data, six ions, for which we were able to identify the major molecules that they came from, were selected for *in vivo* analysis: ions *m/z* 55, *m/z* 61, *m/z* 71, *m/z* 72, *m/z* 87 and *m/z* 137.

**Figure 2:**
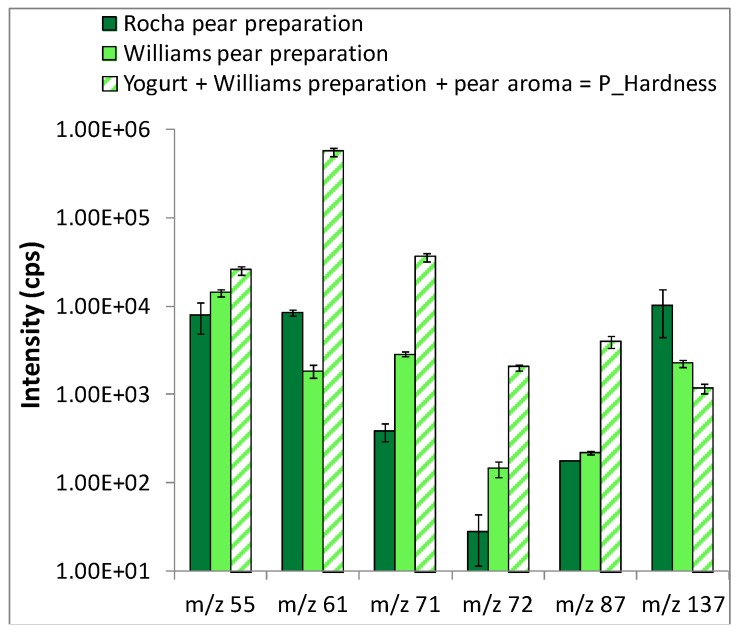
Mean and standard deviation of the intensity of fragments selected for *in vivo* analysis (logarithmic scale). Data were obtained from *in vitro* PTR-MS measurements individually performed in the headspace above pear preparations and above P_Hardness yogurt. The PTR-MS instrument drift tube was thermally controlled (60 °C) and operated with voltage and pressure set at 600.1 (± 0.4) V and 1.9 (± 0.06) mbar, respectively.

Figure 2 compares the intensity of the six fragments selected, obtained from PTR-MS *in vitro* analyses of the headspace above the two fruit preparations separately and above P_Hardness yogurt. On this figure, comparing the intensities of the selected fragments from the Williams pear preparation alone or with the yogurt matrix and pear aroma allows to calculate the part for which the pear preparation is responsible for in the yogurt, knowing that the pear preparation proportion in the yogurt was 15% w/w. For instance, for the fragment *m/z* 71, the pear preparation was responsible for around 60 cps (15% of 380 cps) in the intensity of the yogurt fragment. Then, it appeared clearly that the pear aroma and the yogurt matrix - we cannot distinguish the impact of these two components - contributed more to the total intensity of ion *m/z* 71. The same conclusion was very visible for ions *m/z* 61, *m/z* 71, *m/z* 72 and *m/z* 87, and slightly more nuanced for ions *m/z* 55 and *m/z* 137.

In conclusion, it seems that the flavoring formula and yogurt matrix globally accounted more for the *in vitro* release signals of selected ions than the fruit preparations themselves. This implies that possible aroma release differences observed during *in vivo* measurements could be mainly caused by differences in food oral processes due to fruit piece properties. We therefore based our next interpretations on this first important result.

### 2.3. Impact of the Properties of Fruit Pieces in Yogurts on *In Vivo* Aroma Release

**Figure 3:**
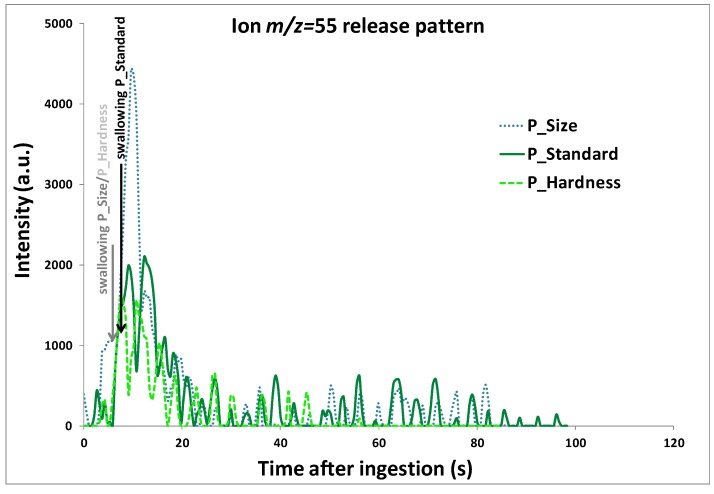
Release kinetics for ion *m/z* 55, corresponding mainly to cis-3-hexenyl butyrate, obtained by *in vivo* PTR-MS measurement with one panelist during the consumption of the three yogurts, P_Standard, P_Size and P_Hardness. The PTR-MS instrument drift tube was thermally controlled (60 °C) and operated with voltage and pressure set at 600.1 (± 0.4) V and 1.9 (± 0.06) mbar, respectively. Time 0 corresponds to the time of product introduction in the mouth.

Despite inter- and intra- individual differences, as well as differences between products on aroma release data, the same release patterns were observed for all panelists and almost all of the ions: a high and rapid increase in aroma release occurred at swallowing for all ions, as already mentioned in the literature [14,38,39], which confirms the large contribution of swallowing to aroma release during food consumption. Afterwards, the intensity rose to a maximum, decreased quite rapidly at first, and then rather slowly until it returned to its initial value. An example of the release pattern for ion *m/z* 55, corresponding mainly to cis-3-hexenyl butyrate, is presented on Figure 3 for one panelist.

For data treatment, some characteristic release parameters were extracted from individual release curves, as described in the Experimental Section. Significant differences observed between P_Standard yogurt and P_Size or P_Hardness yogurts on these parameters are summarized in Table 4 and Table 5, respectively. The release kinetics of P_Standard (6-mm pear pieces) and P_Size (10-mm pear pieces) yogurts were significantly discriminated only on the ∆T20% parameter for ion *m/z* 87: the peak representing diacetyl was the widest in the case of the smallest pear pieces (Table 4), which can be linked to the longest release time of this aroma compound. As diacetyl has a strong affinity with water, it could thus preferentially accumulate in the yogurt matrix which has a high water content, especially as the yogurts studied contained 0% fat. This is why this result can probably be explained by modifications of food oral processing due to changes in the size of fruit pieces.

**Table 4:** Value ratio of characteristic parameters that significantly differ between P_Size and P_Standard products. Characteristic parameters were extracted from *in vivo* release curves (only parameters with significant differences at 5% are shown).

Ion (*m/z*)	87
Parameter	△T20%
Ratio P_Size/P_Standard	0.75
p-value	0.013

**Table 5:** Value ratio of characteristic parameters that significantly differ between P_Hardness and P_Standard products. Characteristic parameters were extracted from *in vivo* release curves (only parameters with significant differences are shown; * trend; * significant at 5%; ** significant at 1%).

Ion (*m/z*)	55		61	72		137				
Parameter	I_max1_	I_max2_	I_max1_	I_max1_	A_1_	A_1_	T_max2_	T20%−	T20%+	T50%+
Ratio P_Hardness/P_Standard	1.32	1.20	1.49	1.28	1.55	1.09	1.20	1.21	1.19	1.20
p-value	0.020*	0.052*	0.020*	0.057*	0.048*	0.023*	0.002**	0.001**	0.002**	0.002**

Concerning the effect of fruit piece hardness, significant differences between P_Hardness and P_Standard yogurts were observed on characteristic parameters for four ions out of the six selected (Table 5). Yogurts containing the hardest pieces (P_Hardness) presented the highest maximal intensity before swallowing (*I*_max1_) for ions *m/z* 55 (*cis*-3-hexenyl butyrate), *m/z* 61 (ester, furaneol) and *m/z* 72 (furaneol), and after swallowing (*I*_max2_) for ion *m/z* 55 (*cis*-3-hexenyl butyrate). Concerning ion *m/z* 55, since no release difference was obtained between the two pear preparations during *in vitro* measurements, the adaptation of oral processing to yogurt type was assumed to be responsible for *in vivo* release differences. For ion *m/z* 72, since *in vitro* and *in vivo* release kinetics presented the same tendency - higher release intensity in the case of hardest pieces - the highest release obtained in the case of yogurt with the hardest pear pieces (P_Hardness) was explained by both pear variety and adaptation of food oral processing to yogurt type. The areas under the curve before swallowing (A_1_) of ions *m/z* 72 and *m/z* 137 were the highest for the P_Hardness product. Ion *m/z* 137 (anisaldehyde) was released later (T_max2_, T20%-, T20%+ and T50%+ higher) in the P_Hardness product than in the P_Standard product. Thus, the hardness of pear pieces seemed to have more impact on *in vivo* aroma release than their size.

### 2.4. Impact of Fruit Piece Structure on the Sensory Characteristics of Yogurt

The sensory evaluation of products was performed using two different methods. First, a sensory profile was used to evaluate the intensity of texture, aroma and taste properties of yogurts. In addition, a Temporal Dominance of Sensations (TDS) test was used to study the dynamic perception of the three yogurts, focusing only on the sequence of dominant aroma and taste perceptions. Both protocols are described in the Experimental Section.

#### 2.4.1. Monitoring Panel Performances

Table 6 summarizes panel performances in terms of repeatability, discrimination power and homogeneity criteria calculated from profile data. Results showed that the panel was repeatable for all attributes, discriminant for 12 attributes out of 14, and homogeneous for the evaluation of nine attributes out of 14. This panel heterogeneity observed for five attributes could be explained either by a non-consensus between panelists while tasting the products (as was the case for three attributes: “fresh green”, “particle size” and “fattiness”), or by a different use of notation scales where some panelists used the whole scale and others just part of it (as was the case for the attributes, “coconut” and “largest piece”). The panel heterogeneity observed on these attributes was taken into account in the data treatment model chosen to analyze the results.

Concerning panel performances using the TDS method, the repeatability index, measured per panelist and per attribute, varied from 22% to 51%, with an average value of 36% over the whole TDS study. This result was judged satisfactory (not shown).

According to these results, the panel was judged reliable enough, allowing analysis of the results and discussion.

**Table 6:** Panel performances determined on profile data and represented by probability values calculated from analyses of variance (Repeatability: ANOVA one factor Repetition per attribute; Homogeneity: ANOVA attribute = Product + Panelist + Product x Panelist; Discrimination power: ANOVA attribute = Product + Panelist + Product x Panelist). Shaded cells indicate that the panel is significantly not reliable for this attribute. (* significant at 5%; ** significant at 1%; *** significant at 0.1%).

	Repeatability (Pr > 0.05 = panel non-significantly different in repeatability)	Homogeneity (Pr < 0.05 = non-homogeneous panel)	Discrimination power (Pr < 0.05 = discriminant panel)
Pear	0.3293	0.0594	0.1241
Citrus fruit	0.2268	0.7217	0.0228
Fresh green	0.4947	0.0199 *	0.0078
Baked butter	0.5573	0.1447	<0.0001
Coconut	0.9614	<0.0001 ***	<0.0001
Caramel	0.774	0.1011	<0.0001
Sweetness	0.3287	0.7995	0.0005
Sourness	0.3469	0.5058	0.293
Homogeneity	0.0627	0.0946	0.0004
Size of the largest piece	0.3981	0.0142 *	<0.0001
Particle size	0.7099	0.0002 ***	0.0023
Fruit piece hardness	0.7885	0.8907	<0.0001
Matrix firmness	0.1114	0.6292	<0.0001
Fattiness of the matrix	0.7285	0.0281 *	<0.0001

#### 2.4.2. Sensory Profile of Yogurts

**Figure 4:**
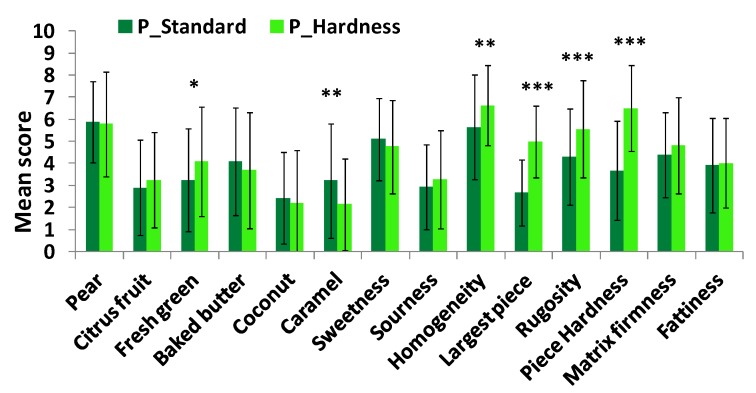
Sensory profiles (mean value across subjects and replicates and standard deviations) of P_Standard and P_Hardness yogurts for each sensory attribute (level of difference significance: * trend; * significant at 5%; ** significant at 1%; *** significant at 0.1%).

Significant differences in aroma perception were highlighted by the panel between P_Standard and P_Hardness (with the hardest pear pieces) yogurts (Figure 4): P_Hardness yogurt was perceived as having a more intense “green” note and less intense "caramel" note than P_Standard yogurt. No significant taste difference was observed between these two yogurts. Regarding texture perception, pear pieces in P_Hardness yogurt were perceived as being significantly harder, rougher, larger and more homogeneous than pear pieces in P_Standard yogurt.

**Figure 5:**
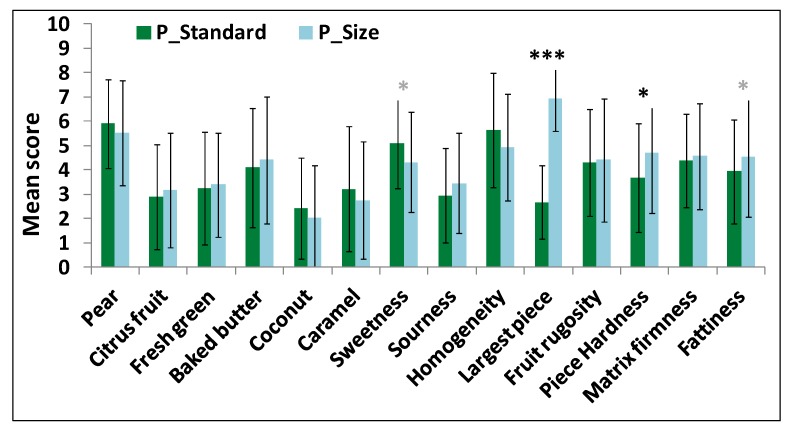
Sensory profiles (mean value across subjects and replicates and standard deviations) of P_Standard and P_Size products for each sensory attribute (level of difference significance: * trend; * significant at 5%; ** significant at 1%; *** significant at 0.1%).

Size difference between fruit pieces was perceived by panelists and induced changes in fruit texture perception: the largest pear pieces (10 mm) were perceived as being significantly harder than the smallest ones (6 mm) (Figure 5). Particle size effect on hardness perception has already been mentioned in the literature in the case of a semi-solid product, even with smallest dispersed particles (2–200 µm) [40]. In this case, effects on roughness and creaminess perceptions in the presence of the largest pieces were also observed. However, in our study, no significant effect was observed on matrix texture perception. One assumption could be that, if particles are as soft as or softer than mucosa, as may be the case for pear pieces, they probably deform upon compression and less contribute to matrix texture perception. Furthermore, P_Size yogurt with the largest pear pieces tended to be perceived as being less sweet and fattier than the P_Standard one (*p* < 0.10). The size of pear pieces did not have a significant impact on aroma perception.

#### 2.4.3. TDS Curves

The mean in-mouth product residence time (all panelists and products), from product introduction into the mouth to the first swallow, was 6.4 ± 4.1 seconds. This short time is in agreement with the semi-liquid texture of this kind of product, for which little mastication is required and which is directly transported from the front of the mouth to the oropharynx by tongue movements [35]. More precisely, the mean time before the first swallow for each of the three yogurts was: 6.0 ± 3.6 seconds for P_Standard, 6.2 ± 4.1 seconds for P_Size and 6.9 ± 4.5 seconds for P_Hardness. These mean values were not significantly different between the three yogurts, meaning that the size and hardness of pear pieces did not influence the residence time of yogurt in the mouth. We can assume that the pear pieces were too small (6–10 mm) or too soft. They were probably directly crushed by compression between the tongue and palate and did not induce modifications of the oral processing time. The mean perception time was 16.4 ± 7.3 seconds, meaning that perception continued ten seconds after swallowing. This persistence time can be explained by the stripping of aroma compounds contained in the residual layer of the food bolus deposited on the pharynx mucosa during the swallow breath [39,41] or to the release after swallowing of odorants first diluted in saliva and adsorbed by the oral mucosa [42,43]. No significant differences between products were observed for this parameter either.

**Figure 6:**
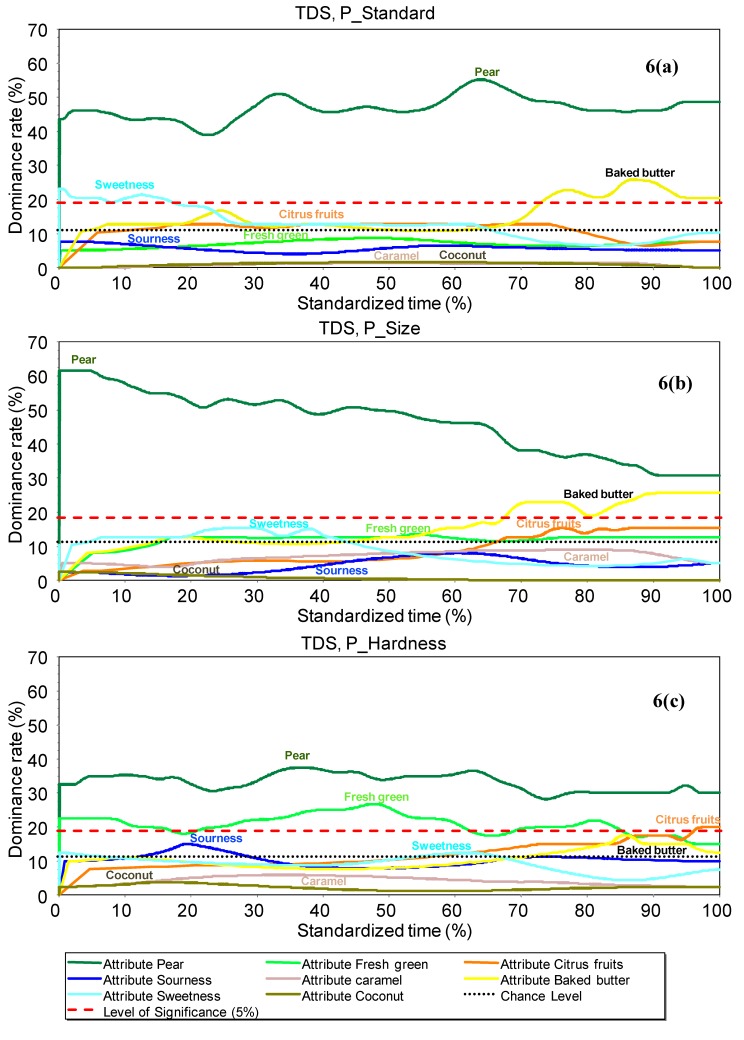
TDS curves: dominance rate (%) over standardized times for the three yogurts. (**a**) P_Standard yogurt; (**b**) P_Size yogurt; (**c**) P_Hardness yogurt. The chance level and the significance level are indicated on each graph. Attributes are considered to be significantly dominant by the panel when they are situated above the significance level.

Figure 6(a–c) present TDS curves for P_Standard, P_Size and P_Hardness products, respectively. First, it can be observed that the "pear" attribute was evaluated as the dominant attribute throughout the perception time for the three yogurts. For the P_Standard yogurt, the “sweetness” attribute was perceived as being dominant at the beginning of perception, and the “baked butter” attribute at the end of the perception time [Figure 6(a)]. Concerning P_Size yogurt (with the largest pear pieces) [Figure 6(b)], the dominance pattern was quite similar to the one of P_Standard yogurt, except for the “sweetness” attribute at the beginning of the perception sequence. We can nevertheless observe that the dominance rate of the “pear” attribute was constant over the consumption of one spoonful of P_Standard yogurt, whereas it decreased in the case of P_Size yogurt. The TDS curves for P_Hardness yogurt (with the hardest pear pieces) showed differences in the dominance rate of “sweetness” and “baked butter” attributes, in comparison to P_Standard: they were no longer significantly dominant (even if they were still above the chance level) [Figure 6(c)]. This is in agreement with profile results, which highlighted that the perception of the “sweetness” attribute tended to be reduced with the presence of the largest pear pieces. This could be explained by a lower sugar diffusion rate in the yogurt matrix due to the physical hindrance caused by the presence of large fruit pieces. However, another hypothesis could be the existence of texture-sapid sensory interactions. The “fresh green” attribute became significantly dominant over almost all of the perception period, which is again in agreement with profile data, and the “citrus fruit” attribute became dominant at the end of the perception period. The “sourness” attribute tended to become dominant at the beginning of perception.

Thus, the hardness of the pear pieces seemed to have more impact on sensory perception, global or dynamic, than their size.

### 2.5. Comparison between *in Vivo* Aroma Release and Sensory Dat

#### 2.5.1. Influence of the Size of Fruit Pieces on Perception and *in vivo* Aroma Release

None of the aroma attributes was perceived differently between yogurts with either 6- or 10-mm size pear pieces. Only the *in vivo* release kinetics of one ion, *m/z* 87, mainly representing diacetyl, showed a short signal persistence when 10-mm pear pieces were present. From the sensory point of view, diacetyl is associated with the sensory note of "baked butter". However, no difference was observed between P_Standard and P_Size yogurts on the perception of this attribute, either on profile or TDS studies. It could be assumed that the difference induced by the size of pear pieces is probably under the panelists' diacetyl threshold detection.

#### 2.5.2. Influence of Hardness of Fruit Pieces on Perception and *in vivo* Aroma Release

Under *in vivo* conditions, the hardest pear pieces led to an increase in the release of almost all of the aroma compounds, especially before swallowing. Ions *m/z* 71 and *m/z* 87 were the only ions among the ones selected that were similarly released, regardless of fruit piece hardness. Concerning ion *m/z* 87, the difference perceived by the panelists on the "baked butter" note cannot be linked to a difference in the release of this specific molecule. We can assume that release differences exist for other molecules present in the product and contribute to this sensory note, but they were not monitored. Moreover, it can also be assumed that the presence of aroma-aroma or texture-aroma sensory interactions could explain this result.

The maximum intensity reached before swallowing (I_max1_) was highest for P_Hardness yogurt containing the hardest pieces, for ions *m/z* 55 (*cis*-3-hexenyl acetate), *m/z* 61 (ester, furaneol) and *m/z* 72 (furaneol). Despite these observations, the panel perceived no difference in “fruity” notes between the two yogurts, perhaps because these release differences were under the detection threshold. The maximum intensity after swallowing (I_max2_) of ion *m/z* 55, related to the “fresh green” perception, was also highest for the P_Hardness product, which can explain the enhancement of the “fresh green” note perception for this product. Release differences observed for ion *m/z* 72, linked to the “caramel” and “cooked fruit” notes, cannot explain why “caramel” perception decreased with fruit piece hardness. It is possible that the predominance of the “pear” note and the increase in the “fresh green” note for the P_Hardness product may have masked the perception of the “caramel” note. 

Ion *m/z* 137 (anisaldehyde) was released later and in a greater quantity before swallowing when the P_Hardness product was eaten compared to the P_Standard product. However, the *in vitro* study showed that this ion was mainly released from pear preparations, especially the one with the Rocha variety (P_Standard). The presence of the hardest pear pieces may have slowed down the diffusion of this volatile compound towards the yogurt matrix and saliva. We can assume that consumers probably naturally adapt their food oral processing behavior to products in order to obtain the most intense sensations, as already reported in previous studies [44]. Further investigation could help to shed light on this hypothesis and to better understand the exact nature of the mechanisms involved.

Finally, no significant taste differences were observed between products, except the loss in the “sweetness” attribute dominance rate in the P_Hardness yogurt. This could be explained by a modification in sugar diffusion properties or by aroma-taste interactions.

## 3. Experimental

### 3.1. Preparation of Flavored Yogurt Samples

Commercial fat-free stirred yogurts (Brassé 0% Auchan, Croix, France) were supplemented with sucrose (7% w/w, Daddy, CristalCo, Paris, France), three types of fruit preparations (15% w/w, Frulact, Gemunde, Portugal) and pear aroma (0.3% w/w, Robertet, Grasse, France). Fruit preparations contained 60% w/w of fruits, 15% w/w of sucrose, and differed by the size and hardness of the fruit pieces (Table 1). To obtain pear pieces with different hardnesses, two pear varieties (Rocha and Williams) were used. The three different yogurts obtained were designated P_Standard, P_Size and P_Hardness. The pear aroma formula was composed of hexyl acetate (0.69% w/w), isoamyl acetate (0.17% w/w), octyl acetate (0.06% w/w), ethyl acetate (0.035% w/w), γ-decalactone (0.03% w/w), *cis*-3-hexenyl acetate (0.03% w/w), geranyl butyrate (0.03% w/w) and pear ester (0.02% w/w) prepared in propylene glycol.

The yogurt base, sucrose, fruit preparation and aroma were successively weighed and mixed together using a food processor (Kenwood, Redmond, WA, USA) equipped with a K-type blade under controlled conditions (minimum power for 30 sec). Yogurt preparation was performed before each sensory or *in vivo* analysis session. The flavoring step was performed between four and six days before consumption to allow aroma stabilization within products. Products were stored at 4 °C. Panelists consumed all yogurts 16–20 days before their expiration date so that they would be in a stable phase of pH and viscosity.

### 3.2. Physico-Chemical Analysis

The hardness of pear pieces in fruit preparations was measured by a puncture test performed at the GMPA laboratory with a TA-XT2 texture analyzer (Stable Micro Systems Ltd., Godalming, UK) fitted with a hemispheric probe of 1 mm in diameter, forced into the fruit with a 2-mm penetration depth. Ten repetitions per type of fruit piece were performed.

The rheological properties and pH of yogurts were measured at each sensory session. The viscoelastic properties of yogurts were measured by an oscillatory test performed with a MCR301 rheometer (Anton Paar GmbH, Graz, Austria), with vane geometry (ST 10-6V-8.8/88, Anton Paar, 4 blades, 10 mm diameter). Measurements from all samples were within the range of linear visco-elasticity. The oscillation frequency sweep test was set at 4 °C with a frequency varying from 10 to 0.1 Hz and a constant deformation of 1%. The measurements were conducted in triplicate. Data obtained were the storage and loss modulus (G' and G'', respectively). These parameters were selected at an angular frequency of 1.58 rad/s to study the results. The pH was measured at 4°C with a pH-metric probe (Mettler Toledo, Viroflay, France).

Real-time nose-space analysis was performed using a High Sensitive Proton Transfer Reaction Mass Spectrometer (PTR-MS) (Ionicon Analytik, Innsbruck, Austria). The PTR-MS instrument drift tube was thermally controlled (60 °C) and operated with voltage and pressure set at 600.1 (±0.4) V and 1.9 (±0.06) mbar, respectively. The E/N value was 153 ± 6 Td and the inlet flow rate was 80.0 mL/min.

For the *in vitro* studies, headspaces above either the water solution with a single aroma compound (molecule concentrations between 100 and 400 mg/kg, 25 g in 0.25-L flasks, Schott AG, Mainz, Germany) or the yogurts (25 g in 0.25-L flasks, Schott), with and without fruit preparation and aroma, were analyzed after an equilibrium period of 24 hours. Measurements were performed in triplicate, at 10 °C, in scan mode from *m/z* 21 to 200 with a dwell time per mass of 50 ms. Fruit preparations with two different pear varieties were also individually analyzed under *in vitro* conditions, following the same protocol. Concerning *in vivo* measurements, the breath of 14 panelists was sampled *via* two inlets of a stainless steel nosepiece placed in both of the panelists’ nostrils. The inlet of the PTR-MS instrument was connected to the sampling device via a 1/16” PEEK™ tube maintained at 60 °C. Measurements were performed in the Multiple Ion Detection mode with a dwell time per mass of 0.5 s, except for *m/z* 21, for which the dwell time per mass was 0.1 s. In addition to the ions previously selected, ion *m/z* 59 (derived from acetone) was monitored to trace panelists’ breath [12]. Moreover, the mass/charge ratios *m/z* 21 (signal for H_3_O^+^) and *m/z* 37 (signal for water clusters H_2_O–H_3_O^+^) were monitored to check instrument performances and cluster ion formation.

*In vivo* analysis was performed following a standardized protocol. Panelists were instructed not to smoke, eat, drink, or use any persistent-flavored product for at least one hour before the session. Each assay lasted 6–7 min. During a given session, panelists had to eat six or seven samples served at 10 °C. Two sessions of 30 min per panelist were planned to obtain three replicates for each product. The room air was first analyzed for 10 s. Then, after positioning the sampling device in the two nostrils, panelists were asked to breathe regularly for 30 s (breath analysis). Panelists were instructed to put one spoonful of yogurt in their mouth and to consume it as they would normally. During all measurements, panelists were asked to keep their mouths closed and to only breathe through the nosepiece. The time of the first swallow was recorded for each panelist. Release data were acquired until release signals returned to their initial intensity. Between each sample, panelists were asked to cleanse their mouth by eating plain crackers and drinking mineral water (Evian, Danone, Evian, France). Panelists’ breath was tested before each new measurement. Samples were coded with three-digit numbers and presented in a sequential monadic way. All the measurements for each panelist were performed within a two-week period.

### 3.3. Sensory Analysis

The sensory evaluation of products was performed using two different methods. First, a sensory profile was used to evaluate the texture, aroma and taste properties of yogurts. In addition, a Temporal Dominance of Sensations (TDS) test was used to study the dynamic perception of the three yogurts, focusing only on aroma and taste perceptions.

Fourteen panelists were recruited at the INRA laboratory according to their motivation and availability to pursue this two-month study with two sessions per week. Potential panelists were screened for self-reported current illnesses, food allergies and intolerances and pregnancy. Each panelist provided written consent to participate in this study. All panelists were trained in preliminary sessions for this specific study. Eight training sessions were designed to help panelists to perceive, recognize and quantify the sensory properties of pear yogurts. Attribute generation was carried out during two specific sessions where a wide range of commercial pear yogurts varying in texture and flavor characteristics was presented. After the generation step, the panelists agreed on a reduced list of attributes related to texture in the mouth, aroma and taste, defined in Table 7. Panelists were then trained to quantify the perception of these attributes on a 10-point unstructured intensity scale, and learned how to use the FIZZ data collection software program (Biosystèmes, Couternon, France, 1999). For the TDS dynamic method, panelists were trained to perceive and describe changes in dominant perception over time. Panelist performances were evaluated prior to data collection, checking individual repeatability and panel consensus.

A sensory profile using the Descriptive Analysis method was performed. The intensity of 14 attributes was assessed on 10-point unstructured intensity scales. Panelists had to successively evaluate taste, aroma and texture of yogurts. They were asked to take homogeneous spoonfuls and to consume them as they would normally. Three replicates per product and panelist were performed in three sessions.

**Table 7:** List of attributes used for the sensory profile and their definitions.

	Attribute	Definition
Taste	Sweetness	Basic taste associated with sucrose
	Sourness	Basic taste associated with lactic acid
Aroma	Baked butter	Aroma perceived by the retronasal pathway associated with diacetyl
	Fresh green	Aroma perceived by the retronasal pathway associated with *cis*-3-hexenol / *cis*-3-hexenyl acetate
	Citrus fruit	Aroma perceived by the retronasal pathway associated with citrus fruit natural extract
	Pear	Aroma perceived by the retronasal pathway associated with hexyl-acetate
	Caramel	Aroma perceived by the retronasal pathway associated with maltol
	Coconut	Aroma perceived by the retronasal pathway associated with γ-octalactone
Texture of fruit	Fruit piece homogeneity	Related to the homogeneity of the size of fruit pieces
pieces	Largest piece size	Size of the largest fruit piece found in the yogurt
	Rugosity	Related to the perception of the size and shape of particles in fruit preparations
	Fruit piece hardness	Related to the force required to compress the fruit pieces between the teeth
Texture of the matrix	Matrix firmness	Related to the force required to compress the product between the tongue and the palate
	Matrix fattiness	Related to the perception of fat in products

The TDS test was performed on the eight taste and aroma attributes. Panelists were instructed to take one spoonful of yogurt and successively select the attributes from the list that triggered his/her attention the most, from product introduction into the mouth (time t=0 when the first attribute was selected) until no sensation was any longer perceived. “I swallowed” and “stop” buttons allowed panelists to indicate the first swallow and the end of evaluation, respectively. Only one attribute could be selected at each time, but panelists were free to select an attribute several times. Attribute order within the list was randomized across panelists, but one panelist always had the same order. For descriptive analyses and the TDS test, yogurts were presented in a sequential monadic way, and a warm-up product was first tested to remind panelists of the test procedure. Three replicates were performed per product and panelist.

Yogurt samples were placed in isothermal plastic cups (40 g/cup) labeled with randomly selected three-digit numbers, covered with a lid and stored at 4 °C until evaluation. Sensory analyses were carried out in individual laboratory booths under a white light, in an air-conditioned room (19 °C). Samples were at approximately 10 °C when they were tested. The presentation order of samples was randomized across panelists to make sure that the run order did not introduce a bias in the results. Panelists were provided with mineral water (Evian, Danone, France) for rinsing and crackers for palate cleaning. The data were directly collected and recorded with FIZZ software (Biosystèmes).

### 3.4. Data Analysis

Data analysis was performed using XLSTAT (Addinsoft, Paris, France, 2009) and Fizz Data Treatment (Biosystèmes).

#### 3.4.1. Aroma Release Parameters

**Figure 7:**
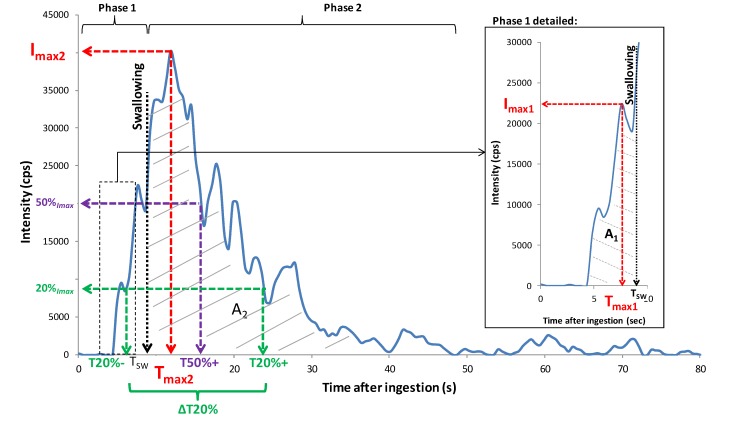
Illustration of parameters extracted for data analysis from an *in vivo* aroma release, obtained for P_Standard yogurt eaten by panelist number 3 (first repetition) and ion *m/z* 61by PTR-MS measurement. Phases 1 and 2 refer to periods before and after swallowing.

For data handling, release curves were divided into three main periods (Figure 7): the phase before the product was put in the mouth (_0_), the oral phase of consumption before swallowing (_1_), and the phase after swallowing (_2_). For each sample, the mean PTR-MS signal measured during phase 0 was subtracted from the PTR-MS signals obtained during the product consumption phases. The following release variables were extracted from each individual release curve and for each phase of product consumption using a software program developed by the laboratory: maximum intensities reached during phases (_1_) and (_2_) of in-nose analyses (*I*_max1_ and *I*_max2_), times necessary to reach *I*_max1_ and *I*_max2_ (T_max1_ and T_max2_), taking starting time (T_0_) as the moment when the spoonful of yogurt was introduced into the mouth, and areas under the curve (A_1_ and A_2_). Times necessary to reach 20% of *I_max_* before and after T_max_ (T20%- and T20%+) and times necessary to reach 50% of I_max_ after T_max_ (T50%+) were also recorded. They represent the in-mouth release rate and release persistence, respectively. Parameter △T20% was calculated as the time difference between T20%− and T20%+ and represents the peak width. The time that elapsed between yogurt introduction into the mouth and swallowing (T_SW_) was also recorded. All these parameters were subjected to a two-way analysis of variance (ANOVA) with interactions (product, panelist) to determine significant differences between the products and the panelists.

#### 3.4.2. Sensory Analysis

Panel performances were monitored by evaluating the repeatability, discrimination power and homogeneity of panelists and of the panel. For TDS data, the repeatability index represents panelist consistency in their evaluation of a product. It corresponds to the percentage of common sensation sequences measured between the three replicates of each product (R1, R2 and R3) for each subject. First, for each subject and for each yogurt, the percentage of common sequences between each combination was calculated (R1/R2, R1/R3 and R2/R3) according to the formula below. The sequences were constructed by 250 ms-time periods. To calculate the percentage of common sequence between two sequences, they were compared chronologically by 250 ms-time period. Three percentages were obtained by combination Yogurt × Subject:


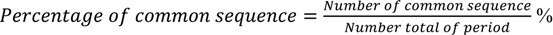
(1)

Then, the average of these three percentages by combination Yogurt ° Subject was calculated. Three percentages were obtained for each subject (one per yogurt). Finally, the average percentage got for the panel was calculated.

#### 3.4.3. Profile Data Analysis

Student t-tests and two-way analyses of variance with interaction (ANOVA) using product and panelist as factors were performed for each attribute to determine if products could be discriminated by the trained panel. When differences were observed (*p* < 0.05), mean scores were compared using the Newman-Keuls multiple comparison test.

#### 3.4.4. TDS Curves

TDS curves for each product were constructed with the panel dominance rate (%) calculated per product for each attribute and at each point in time, as described by Pineau *et al.* [10]. The end of the curve represents the time at which perception ends (T_end_). In order to compare products, TDS curves were also determined using a standardized time scale, from 0% (beginning of consumption) to 100% (end of perception), as described in the literature [45].

## 4. Conclusions

To conclude, the size of pear pieces in yogurt, in the studied range, had only a few effect on aroma release and perception. Concerning the hardness of pear pieces, it significantly influenced aroma release and globally led to an increase in the exposure to aroma stimuli (intensity and duration) for ions that were monitored during *in vivo* measurements. Some of these differences were even perceived by the panelists, inducing modifications of the global and dynamic sensory properties of yogurts.

Aroma release differences were observed between yogurts with different pear structure. An adaptation of food oral processing to fruit structure was assumed, despite a non significant difference in residence time of yogurt in the mouth. This result could be explained by the short product in-mouth residence time in the case of yogurt consumption, but also because, even if the fruit piece hardness was significantly different (and this difference was significantly perceived by the panel), the pear pieces were rather small (6 mm), so that mixed in the yogurt matrix they did not required a real mastication. 

In this study, the yogurt texture (fruit pieces) and the oral processing varied at the same time, which make difficult to determine the respective contribution of these two factors. A free consumption protocol was used by panelists during *in vivo* measurements to be as close as possible to the real conditions of consumption, inducing various oral processing adapted by each panelist to each product. Previous intern studies [46] showed that higher product discrimination was observed when free protocols of consumption were used. But to eliminate potential cross-over effects between product properties and consumption protocol, it could be interesting to compare results obtained with free or controlled consumption protocols.

Finally, this study showed that fruit preparation could be an interesting formulation factor to enhance exposure time to stimuli and thus modify food oral processing. These results could be taken into account to formulate new products with both nutritional and sensory criteria. To further improve our understanding, the impact of these formulation factors on digestion, self-regulation and food intake could be investigated since it was shown that longer exposure time of olfactory receptors to aroma stimuli and oral sensory exposure can lead to enhanced feelings of satiation and may ultimately contribute to a decrease in food intake [4,47].
